# Supplementary material for: Evaluation and Characterization of the Insecticidal Activity and Synergistic Effects of Different GroEL Proteins from Bacteria Associated with Entomopathogenic Nematodes on Galleria mellonella
Source: Toxins (Basel). 2023 Oct 24;15(11):623. doi: 10.3390/toxins15110623 (PMC10674725; doi:10.3390/toxins15110623)
Supplement: Supplementary file 1 [file toxins-15-00623-s001.zip › toxins-2666461-supplementary.pdf]

toxins

MDPI

Supplementary Materials

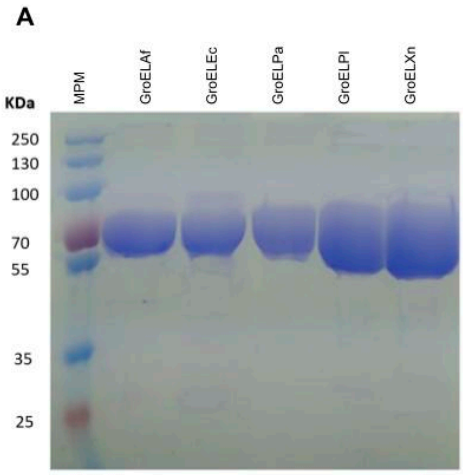

B

Strain	Protein	Yield (µg/ml)
A.faecalis MOR02	GroELAf	1402.93
E.coli DH5α	GroELEc	1327.71
P.aeruginosa NA04	GroELPa	1389.81
P.luminescens HIM3	GroELPl	1825.32
X.nematophila SC 0516	GroELXn	1907.67

Figure S1. SDS-PAGE of the different GroEL proteins expressed in pET28a and purification yields. (**A**) MPM: molecular weight markers in lane 1. 15 ul of sample per protein were placed in lane 2: GroElAf, lane 3: GroELEc, lane 4: GroELPa, lane 5: GroELPl and lane 6: GroELXn; (**B**) Table with the yields obtained from the purification of GroEL proteins.

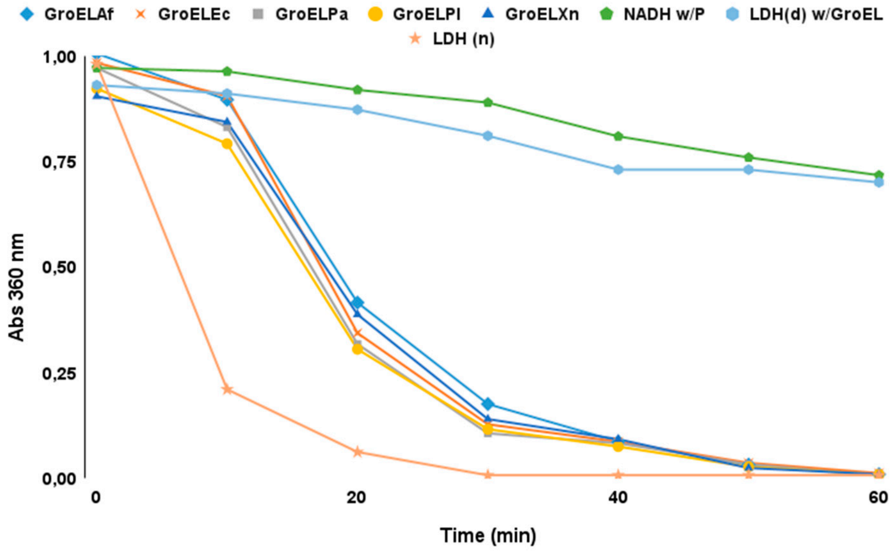

Figure S2. Time course of reactivation of heat denatured Lactate Dehydrogenase (LDH) promoted by GroEL chaperonin. A five-fold excess of the molar concentration of GroEL (50 µM) with respect to the enzyme was added to the refolding reaction. The reaction mixture without protein only NADH compound (NADH w/P), denatured LDH without GroEL ($LDH_{(d)}$ w/GroEL, as well as native LDH ($LDH_{(n)}$) were used as controls.

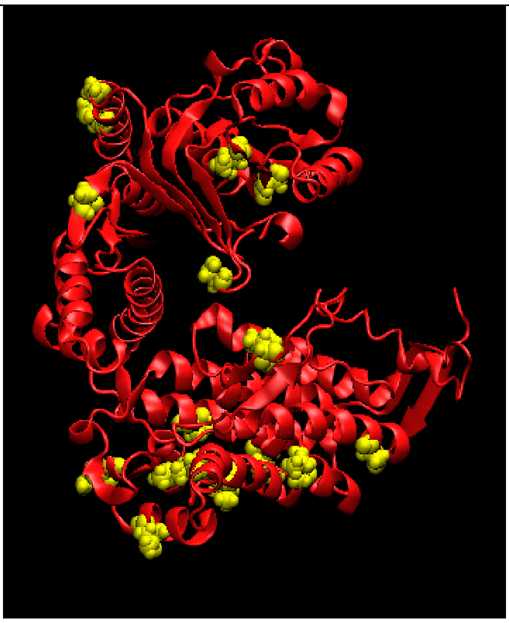

Figure S3. 3D representation of the GroELXn subunit on which the 15 unique substitutions (in yellow) were mapped to their structural homologs. The structure was obtained from the protein sequence in the ITASSER online server and visualized with the VMD 1.9 program.
